# Efficacy and safety of conversion to monotherapy with eslicarbazepine acetate in adults with uncontrolled partial-onset seizures: a historical-control phase III study

**DOI:** 10.1186/s12883-015-0305-5

**Published:** 2015-03-28

**Authors:** Mercedes P Jacobson, Ladislav Pazdera, Perminder Bhatia, Todd Grinnell, Hailong Cheng, David Blum, on behalf of the study 046 team

**Affiliations:** Department of Neurology, Temple University School of Medicine, Philadelphia, PA USA; CTC Rychnov nad Kněznou s.r.o, Rychnov nad Kneznou, Czech Republic; Neuro-Pain Medical Center, Fresno, CA USA; Sunovion Pharmaceuticals Inc., Marlborough, MA USA

**Keywords:** Antiepileptic, Eslicarbazepine acetate, Partial onset seizures, Epilepsy, Monotherapy

## Abstract

**Background:**

Eslicarbazepine acetate (ESL, Aptiom®) is a once-daily (QD) anticonvulsant, approved as adjunctive treatment of partial-onset seizures (POS). It is extensively converted after oral administration to eslicarbazepine, and is believed to exert its effect through inhibition of voltage-gated sodium channels. The possible role of ESL as monotherapy to treat POS has not yet been established.

**Methods:**

This study was an 18-week, multicenter, randomized double-blind trial of gradual conversion to ESL monotherapy in adults with POS not well controlled by 1–2 antiepileptic drugs (AEDs), using historical data as the control. The study comprised an 8-week baseline period, a 2-week titration period, a 6-week AED conversion period, a 10-week monotherapy period, and either a 1-week taper period or optional entry to an open-label extension study. The primary endpoint compared the Kaplan–Meier (KM)-estimated 112-day exit rate with a threshold value calculated from the historical controls.

**Results:**

There were 172 randomized patients; 154 (90%) entered the AED conversion period and 121 (70%) completed the study. The KM-estimated exit rates [confidence interval (CI)] were 15.6% [8.1–28.7%] for ESL 1200 mg, and 12.8% [7.5–21.5%] for ESL 1600 mg. The upper limits of the 95% CI KM-estimates were below the pre-specified threshold for historical control of 65.3%, indicating that ESL was efficacious in reducing seizure-related exits, compared with historical control. During the 18-week double-blind treatment period, median reductions in standardized seizure frequency occurred with ESL 1200 mg (36.1%) and ESL 1600 mg (47.5%). The responder rates (a 50% or greater reduction in seizure frequency from baseline) during the 18-week double-blind period and the monotherapy period, respectively, were 35.2% and 38.9% for ESL 1200 mg, and 46.0% and 46.0% for ESL 1600 mg. The overall adverse event profile was consistent with the known safety profile of ESL.

**Conclusions:**

These findings indicate that ESL monotherapy (1200 and 1600 mg QD) was efficacious and well tolerated in this study.

**Trial registration:**

NCT01091662; EudraCT No. 2010-018684-42.

**Electronic supplementary material:**

The online version of this article (doi:10.1186/s12883-015-0305-5) contains supplementary material, which is available to authorized users.

## Background

The incidence of epilepsy worldwide has been estimated to be between 16 and 51 per 100,000 (age-adjusted) [1]. Antiepileptic drugs (AEDs) are the mainstay of epilepsy treatment [2]. AED monotherapy is generally preferred to adjunctive therapy, due to the greater risk of adverse events (AEs) and adverse drug interactions with combination therapy [3]. Moreover, AED monotherapy is valuable for certain types of patients, including women, the elderly, and those with co-morbid conditions, for whom AED toxicity and drug interactions may have additional consequences [4,5]. There is an unmet clinical need for new AEDs that are both effective and well tolerated [4], for use as monotherapy in the treatment of patients with epilepsy.

Eslicarbazepine acetate (ESL) is an AED; a member of the dibenzazepine family. ESL is structurally different from the other dibenzazepines, carbamazepine (CBZ) and oxcarbazepine (OXC) [6]. Following oral administration, ESL undergoes first-pass hydrolysis in the liver, being rapidly metabolized to the active metabolite, eslicarbazepine [7,8]. Eslicarbazepine inhibits sodium currents by binding to voltage-gated sodium channels (VGSCs) and stabilizing the inactivated state of the channel and, compared with CBZ, shows a higher relative affinity for the inactive versus the resting state of VGSCs [9]. Eslicarbazepine has an apparent half-life of 13–20 hours in plasma [10] and 20–24 hours in cerebrospinal fluid [11], which supports once-daily (QD) dosing.

The efficacy and safety of ESL as adjunctive therapy in adults with partial-onset seizures (POS) is well established [12-15]. ESL (Aptiom®) was approved by the European Medicines Agency in 2009 (as Zebinix®) for adjunctive therapy in adults with POS with or without secondary generalization [16], and by the US Food and Drug Administration in November 2013 as an adjunctive treatment for POS [10]. The potential role of ESL as monotherapy for POS has not previously been investigated.

Here we report the results of a phase III clinical trial that evaluated the efficacy and safety of ESL as monotherapy for patients with POS not well controlled by their current AEDs. Efficacy was evaluated by comparison with a historical control group (an approach advocated by French et al., 2010 [17]). Some previous studies (e.g., for zonisamide and pregabalin) used an active-control non-inferiority design to investigate the efficacy of AEDs as monotherapy [18,19]. Such trials allow direct comparison with active treatment, but are susceptible to false positive findings of equivalence [20] if the placebo response rate is high, or if subjects with unusually low risks of recurrence are recruited. Trials designed to show statistical superiority over a historical control can provide complementary evidence of efficacy. This trial uses an identical protocol to study 045, which was performed in a North American population [21].

## Methods

The study (ClinicalTrials.gov identifier: NCT01091662; EudraCT No. 2010-018684-42) was performed between June 2010 and November 2012, and was designed and conducted in accordance with all relevant regulations and guidelines. The protocol was approved by the Institutional Review Board (Copernicus Group IRB) and an independent ethics committee. Written informed consent was obtained from all patients.

### Patients

Table 1 shows the key inclusion and exclusion criteria. Eligible patients were 16 to 70 years of age, with partial epilepsy, as defined by the International League Against Epilepsy [22], a history of seizures, an electroencephalogram consistent with partial epilepsy, and absence of a progressive structural abnormality, as shown by a computerized tomography or magnetic resonance imaging scan within the previous 10 years. During the 8 weeks prior to screening, patients must have had at least four POS, and no seizure-free period ≥4 weeks in duration. They must also have been receiving stable doses of 1–2 AEDs for 4 weeks prior to screening. Those receiving two AEDs at screening were enrolled if at least one AED was not a sodium channel blocker (phenytoin, CBZ, OXC, lamotrigine) and at least one was not in the upper dose range (more than two-thirds of the defined daily dose; Table 2).

**Table 1:** **Patients; inclusion and exclusion criteria**

**Major inclusion criteria**	**Major exclusion criteria**
• Male and female patients aged ≥16 to ≤70 years with partial epilepsy (defined by the International League Against Epilepsy, 1981 [19]) and a medical history of seizures.	• Patients with only simple partial seizures without a motor component.
	• Presence of generalized seizure syndromes.
• Absence of confounding factors (e.g. pseudoseizures, syncope).	• History of pseudoseizures.
• Documented electroencephalography recording consistent with partial-onset epilepsy and documented computerized tomography or magnetic resonance imaging scan showing absence of a progressive structural abnormality (within 10 years prior to screening).	• Current seizures relating to acute medical illness, or seizures secondary to metabolic, toxic or infectious disorder or drug abuse.
• ≥4 partial onset seizures 8 weeks prior to screening with no 4-week seizure-free period.	• Status epilepticus within 2 years prior to screening.
• Treatment with a stable dose of 1–2 AEDs in the 4 weeks prior to screening. In the situation where a patient was receiving two AEDs at screening, the patient was enrolled if:	• Seizures only occurring in a cluster pattern.
- One of the two AEDs was not one of the following sodium channel blockers: phenytoin, carbamazepine, oxcarbazepine, or lamotrigine; and	• Psychiatric history, including major depressive episode within 6 months, active suicidal plan or intent within the past one month, history of suicide attempt, significant psychiatric disorder, or alcohol or substance abuse within 2 years.
- The second of the 2 AEDs was not being dosed in the upper dose range (defined as greater than approximately two-thirds of the defined daily dose^*^).	
• In elderly patients (65–70 years), no additional/potential health complications.	

^*^Defined daily doses of AEDs are shown in Table 2.

*AED* = antiepileptic drug.

**Table 2:** **Defined daily dose (DDD) for concomitant AEDs**

**AED**	**Approximately 2/3 DDD**	**Adult DDD**
Carbamazepine^*^	700 mg	1000 mg
Ethosuximide	800 mg	1250 mg
Felbamate	1600 mg	2400 mg
Gabapentin	1200 mg	1800 mg
Levetiracetam	1000 mg	1500 mg
Lamotrigine^*^	200 mg (200 mg with enzyme inducers; 100 mg with valproate)	300 mg (400 mg with enzyme inducers; 150 mg with valproate)
Lacosamide^†^	300 mg	200–400 mg
Oxcarbazepine^*^	700 mg (1000 mg with enzyme inducers)	1000 mg (2000 mg with enzyme inducers)
Phenobarbital	70 mg	100 mg
Pregabalin	200 mg	300 mg
Phenytoin^*^	200 mg	300 mg
Primidone	800 mg	1250 mg
Tiagabine	20 mg (30 mg with enzyme inducers)	30 mg (60 mg with enzyme inducers)
Topiramate	200 mg (300 mg with enzyme inducers)	300 mg (600 mg with enzyme inducers)
Vigabatrin	1400 mg	2000 mg
Valproate	1000 mg	1500 mg
Zonisamide	140 mg (200 mg with enzyme inducers)	200 mg (400 mg with enzyme inducers)

Note: The DDD is the assumed average maintenance dose per day for a drug used for its main indication in adults [32].

^*^Sodium channel blockers; ^†^Adult DDD information from product label.

DDD = defined daily dose; AED = antiepileptic drug.

### Procedures

The study used a “withdrawal to monotherapy” design. The effects of active treatment were compared with those of a virtual placebo historical control group, defined by French et al. [17]. Use of a historical control (based on the exit rates in eight different monotherapy studies of similar design and duration to the current study, including randomization as done in the original studies) eliminated the need for a placebo group, meaning that all patients received active treatment. Patients were screened at 50 investigational sites (25 US; 25 ex-US). Those who successfully completed screening procedures at 41 investigational sites (18 US and 23 ex-US) entered an 8-week baseline period for assessment of seizure frequency. Patients meeting all inclusion criteria were randomized 2:1 to receive ESL 1600 or 1200 mg QD for 18 weeks. 1200 mg QD was selected as the highest dose to have been shown to be effective and well tolerated in adjunctive trials. Based on evidence with other AEDs used as monotherapy, it was postulated that the higher dose of 1600 mg QD would provide greater efficacy, and would be tolerated in the monotherapy setting. Randomization was performed using an interactive voice-response system to associate each patient with double-blind clinical trial material (‘kits’) and a randomization number. The randomization list was prepared by a third party using a random number generator, following a permutated-block design (block size = 6). Placebo capsules to match over-encapsulated ESL 400 mg and 600 mg were supplied to maintain the blind.

**Figure 1:**
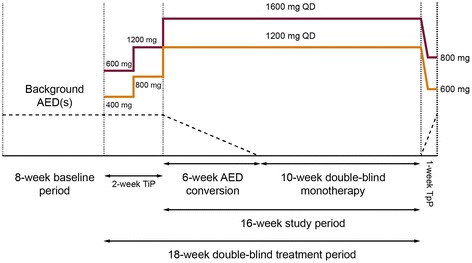
**Study design.** Patients randomized to 1600 mg QD of study drug titrated from 600 to 1200 to 1600 mg QD over the 2-week titration period and down-titrated from 1600 to 800 mg QD after 3 days of the start of the 1-week taper period. Patients randomized to 1200 mg QD titrated from 400 to 800 mg to 1200 mg QD over the 2-week titration period and down-titrated from 1200 to 600 mg QD after 3 days of start of taper period. Patients started other AEDs during the taper period. AED = antiepileptic drug; QD = once daily; TiP = titration period; TpP = taper period.

Patients randomized to 1200 mg ESL QD received 400 mg QD during Week 1 and 800 mg QD during Week 2; those patients randomized to 1600 mg ESL QD received 600 mg QD during Week 1 and 1200 mg QD during Week 2 (Figure 1). The doses of AEDs being used at baseline were then gradually reduced (by 50% over the next 3 weeks, and to zero over the subsequent 3 weeks). When two AEDs were being taken, both drugs were withdrawn concurrently (Figure 1). Throughout the 6-week conversion period, and for the next 10 weeks, patients continued to receive their allocated ESL dose (1200 or 1600 mg QD). After the first 3 days of the 1-week taper period, ESL doses were down-titrated from 1200 to 600 mg QD, and from 1600 to 800 mg QD. Patients who completed the first 3 weeks of double-blind treatment, and who subsequently completed, discontinued, or exited for reasons other than safety were eligible to participate in a long-term safety open-label extension study (long-term data will be presented separately).

### Assessments

The patients were evaluated in clinic at baseline, at randomization, and after the start of ESL dosing, at Weeks 1, 2, 5, 8, 11, 14, 18, and 19. Additionally there was telephone contact at Weeks 3, 4, 6, 7, 9, 10, 12, 13, 15, 16, and 17.

#### Primary endpoint

Seizure data were collected using a seizure diary, completed daily by the patients, throughout the study. Investigators reviewed the seizure diaries with the patients during clinic visits, to decide whether or when patients had met one of the five prospectively defined exit criteria (signifying worsening seizure control) before the end of the 16-week study period (shown in Figure 1): one episode of status epilepticus; one secondary generalized partial seizure (for patients who did not have generalized seizures in the 6 months before screening); a doubling of any consecutive 28-day seizure rate, compared with the highest such rate during the baseline period; a doubling of any consecutive 2-day seizure rate, compared with the highest such rate during the baseline period (or 3 seizures in 2 days, if the highest rate during the baseline period was 1 seizure per 2 days); worsening of seizures, or an increase in seizure frequency considered serious or requiring intervention, as judged by the investigator. The exit criteria were developed on the basis of historical and baseline data. For the two criteria related to doubling of seizure rates, the analysis of the primary endpoint was conducted using seizure rates calculated by the study statisticians. The primary efficacy endpoint, i.e., the exit rate for a treatment arm, is defined as the proportion of patients meeting at least one of the above exit criteria during the 16-week (112-day) study period.

Treatment was considered effective (and the null hypothesis was rejected) if the upper 95% confidence limit (UCL) for the exit rate (estimated using Kaplan–Meier methodology) was below the lower limit of the pre-specified prediction interval (65.3%) calculated from historical controls [17].

#### Secondary endpoints

The key secondary endpoint was the seizure-free rate (%) during the 10-week monotherapy period. Additional secondary endpoints included: seizure-free rate during the last 4 weeks on ESL monotherapy; completion rate (patients completing 18 weeks of double-blind treatment [%]); completion rate for the 10-week monotherapy period (%); change in standardized seizure frequency (between the baseline period and the 18-week double-blind period or the 10-week monotherapy period); responder rate (proportion of patients with ≥50% reduction in seizure frequency, between the baseline period and the double-blind period or the monotherapy period); change in standardized seizure frequency by seizure type (between the baseline and monotherapy periods); responder rate by seizure type; change from baseline in Quality Of Life In Epilepsy (QOLIE-31) scores; change from baseline in Montgomery–Asberg Depression Rating Scale (MADRS) scores (for all patients, and for patients with a MADRS score ≥14 at randomization).

#### Safety and tolerability

AE reports were collected from patients at each clinic visit, from the time they provided informed consent, to the end of the study. AEs were coded according to their respective system organ class and preferred term using Medical Dictionary for Regulatory Activities (MedDRA) version 13.1. The occurrence and intensity (mild, moderate, or severe) of AEs were recorded by the investigators. Serious AEs were reported separately; classification of AEs as “serious” was at the judgment of the investigator. Treatment-emergent AEs (TEAEs) were defined as those AEs that occurred on or after the first dose of the study drug. Summary statistics for TEAEs were calculated for each study period (titration, AED conversion and ESL monotherapy periods) for both treatment groups and overall.

### Statistical analyses

For the primary efficacy endpoint (exits based on seizure criteria), a sequential testing procedure for type 1 error control was pre-specified and implemented. The first comparison was for the ESL 1600 mg group versus historical control, followed by the ESL 1200 mg group versus historical control and then a comparison between the two groups via log-rank testing. French et al. [17] calculated a 95% prediction interval based on the exit rates reported in historical trials. The lower bound of the prediction interval for a single study is an exit rate of 65.3% at 112 days [17]. Thus, if the 95% UCL for a treatment group was <65.3%, then the null hypothesis (that the exit rate for the test group equals the combined exit rate derived from the historical controls) could be rejected. The exit rate was estimated using Kaplan–Meier methods, using the time to exit observed for each patient.

Patients were censored if they withdrew from the study for reasons other than meeting the exit criteria, or if they completed 112 days of treatment without meeting the exit criteria. The censor rate for early withdrawal reported in historical control trials was ~10%. Consequently, the protocol specified that if the withdrawal rate for reasons other than meeting exit criteria exceeded 10%, the additional withdrawals would be reassigned as exits through random sampling. A secondary analysis of the primary endpoint was performed, in which censored patients were not reassigned as exits, even if the dropout rate was >10%.

An analysis of the exit rate was also performed, according to use of AEDs at baseline (for AEDs used in ≥20% of patients), using the same method as above. The potential effects of covariates on exit rate were assessed using Cox proportional hazards regression models.

The statistical analyses of the secondary efficacy endpoints and safety endpoints are described in the Additional file 1: Appendix S1. All statistical procedures were performed using SAS version 9.2 or higher. All statistical tests were two-sided, and the type I error rate was fixed at 0.05.

#### Determination of sample size

Patients were randomized in a 2:1 ratio to either ESL 1600 or 1200 mg QD. The exit rate is assumed to be 54% for the 1600 mg QD arm, corresponding to a weekly hazard rate of 4.85%. This exit rate represents a 39% reduction in the exit hazard rate based on the lower end of the 80% prediction interval established for replication in two clinical studies (72.2%; weekly hazard rate of 8%) and a 20% reduction in the exit hazard rate based on the lower end of the 95% prediction interval established for a single clinical study (65.3%; weekly hazard rate of 6.62%). For ~114 patients randomized to ESL 1600 mg, and assuming a 10% discontinuation rate without observed exit events, there was ≥95% chance that the UCL for the observed exit rate would fall below 72.2%, and a 70% chance that the UCL would fall below 65.3%. Assuming an early dropout rate of 20%, ~200 patients were required to enter the baseline period to achieve a minimum of 171 randomized patients. Fewer patients were enrolled at 1200 mg, as the lower dose arm was included for blinding purposes, and to assess the possibility of a dose–response relationship (although the study was not adequately powered to detect a statistical difference between doses).

#### Study populations

The intention-to-treat (ITT) population consisted of all randomized patients who received at least one dose of the study drug; the ITT population was used to evaluate patient disposition, baseline demographics and characteristics, and safety outcomes. Primary and secondary efficacy analyses were based on the efficacy (EFF) population (all ITT patients who entered the AED conversion period). An additional analysis of the primary efficacy endpoint was conducted for the per-protocol population (patients in the EFF population without important protocol deviations).

## Results

### Patient demographics and baseline characteristics

Overall, the ITT population included almost equal proportions of males and females (Table 3). Their median age was 36.5 years; the majority were white (93.0%) and living outside the US (75.0%). A small minority (4.7%) were Hispanic or Latino. At study entry, most patients (64.5%) were receiving one AED. CBZ and valproic acid were the most commonly used AEDs during the baseline period (by ≥20% of patients; Additional file 1: Table S1). Benzodiazepines were used intermittently (emergency use) by 1.7% of patients taking ESL 1200 mg and 2.6% of patients taking ESL 1600 mg. During the baseline period, the mean maximum 2-day seizure rate was 2.4 and the mean maximum 28-day seizure rate was 10.5. Demographics and baseline characteristics of the EFF and per-protocol populations were comparable to that of the ITT population (data not shown).

**Table 3:** **Demographic and clinical characteristics of the ITT population** ^*^

**Characteristic**	**ESL 1200 mg**	**ESL 1600 mg**	**Total**
	**(n = 58)**	**(n = 114)**	**(n = 172)**
Age, years; median (range)	37.0 (16–60)	35.5 (16–65)	36.5 (16–65)
Gender, male; n (%)	31 (53.4)	52 (45.6)	83 (48.3)
Race; n (%)			
White	53 (91.4)	107 (93.9)	160 (93.0)
Black or African American	5 (8.6)	1 (0.9)	6 (3.5)
Other	0	6 (5.3)	6 (3.5)
Region; n (%)			
US	15 (25.9)	28 (24.6)	43 (25.0)
Non-US	43 (74.1)	86 (75.4)	129 (75.0)
BMI, kg/m^2^; median (range)	25.6 (17–59)	24.3 (17–51)	24.7 (17–59)
Maximum consecutive 2-day baseline seizure rate			
Mean ± SD	2.2 ± 1.84	2.5 ± 1.46	2.4 ± 1.60
Maximum consecutive 28-day baseline seizure rate			
Mean ± SD	9.2 ± 6.72	11.1 ± 7.94	10.5 ± 7.59
Baseline AEDs used by ≥20% patients^†^; n (%)			
Carbamazepine	22 (37.9)	27 (23.7)	49 (28.5)
Valproic acid^‡^	12 (20.7)	39 (34.2)	51 (29.7)
Number of AEDs at baseline^†^; n (%)			
1	41 (70.7)	70 (61.4)	111 (64.5)
2	17 (29.3)	44 (38.6)	61 (35.5)

^*^At entry to the baseline period.

^†^An AED was considered to be used at baseline if it was started at any time prior to first dose of study drug and continued into the titration period.

^‡^Includes all forms of valproic acid (ergenyl chrono, valproate semisodium, valproate sodium and valproic acid).

Note: Percentages are calculated based on the number of patients with non-missing data in the ITT population in each column.

*ITT* = intention-to-treat; *ESL* = eslicarbazepine acetate; *US* = United States; *BMI* = body mass index; *SD* = standard deviation; *AED* = antiepileptic drug.

Treatment groups were generally well balanced in terms of demographics and baseline characteristics (Table 3), but there was a greater proportion of Black/African-American patients in the ESL 1200 mg group (8.6%) than in the ESL 1600 mg group (0.9%).

### Patient disposition and adherence to study drug

**Figure 2:**
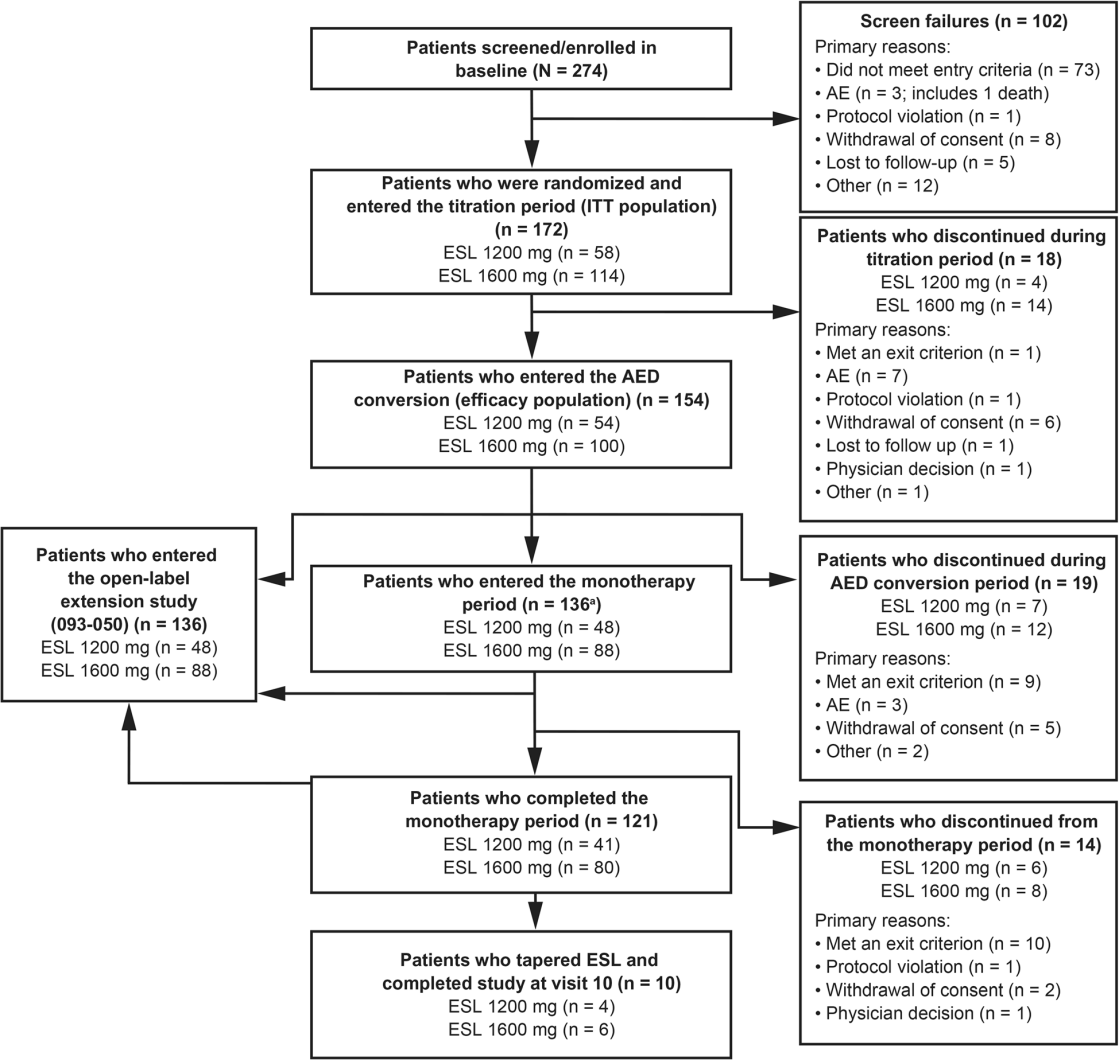
**Patient flowchart**. ^*^One patient met an exit criterion during the AED conversion period and was discontinued from the study, but returned for the subsequent visits 6 and 7, and so was also counted as having entered the monotherapy period. AE = adverse event; ITT = intention-to-treat; ESL = eslicarbazepine acetate; AED = antiepileptic drug.

Of the 274 patients screened, 172 were randomized to study treatment and began the titration period (the ITT population; Figure 2). Eighteen patients discontinued during ESL titration (ESL 1200 mg, n = 4; ESL 1600 mg, n = 14), and consequently, 154 patients began the conversion to monotherapy (AED conversion) period (the EFF population). Nineteen patients discontinued during the conversion period (ESL 1200 mg, n = 7; ESL 1600 mg, n = 12). One patient who met an exit criterion during the conversion period was discontinued from the study, but then attended two further visits, so was also counted as having entered the monotherapy period, producing a total of 136 patients entering the monotherapy period. A total of 121 patients completed the monotherapy period (6 patients discontinued from the ESL 1200 mg group and 8 from the ESL 1600 mg group).

During the double-blind period, 90% of patients had 80–120% adherence to the study drug, as evaluated by pill counts.

### Efficacy

#### Primary endpoint

**Figure 3:**
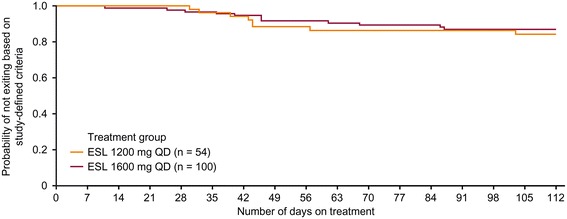
**Kaplan–Meier plot of time to exit (EFF population).** EFF = efficacy; ESL = eslicarbazepine acetate; QD = once-daily.

**Figure 4:**
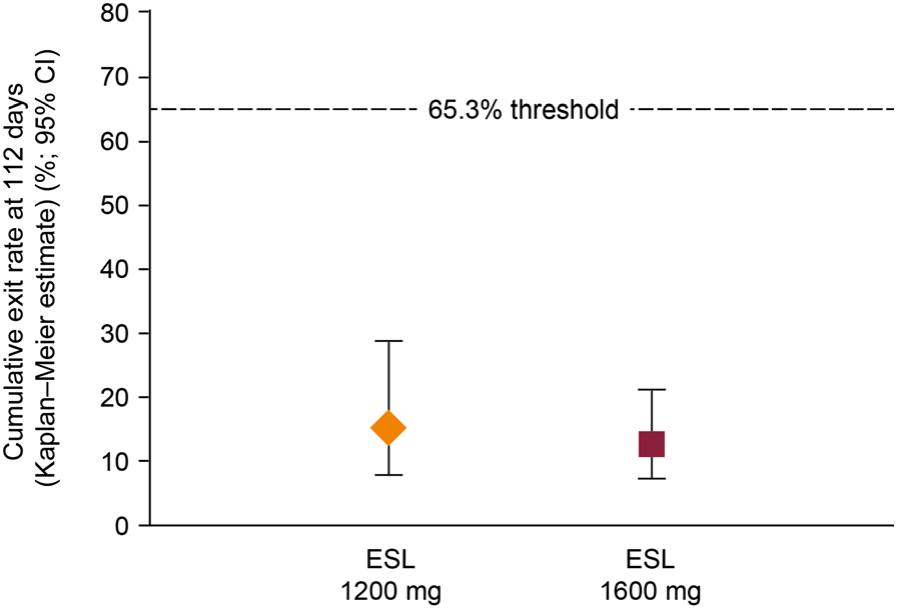
**Kaplan–Meier estimates of exit rate at 112 days (EFF population).** EFF = efficacy; CI = confidence interval; ESL = eslicarbazepine acetate.

During the 16 week study period, 19 patients (12.3%) met one of the five predefined exit criteria (ESL 1600 mg, n = 12; 1200 mg, n = 7). Patients either met the exit criteria during the AED conversion period (n = 9) or the monotherapy period (n = 10); one patient who met an exit criterion during the titration period was not included in the efficacy analysis. One patient who dropped out was reassigned as an exit in the ESL 1200 mg arm. The Kaplan–Meier-estimated exit rate was 12.8% [95% CI 7.5–21.5%] for ESL 1600 mg and 15.6% [8.1–28.7%] for ESL 1200 mg (Figure 3). Thus the UCLs for the Kaplan–Meier exit rates (1600 mg, 21.5%; ESL 1200 mg, 28.7%) were both below the 65.3% threshold calculated from the historical controls (Figure 4), demonstrating that the exit rates for the two ESL doses were significantly lower (signifying fewer seizure exits) than those observed for the historical controls. The exit rates were similar for both ESL dose groups (log-rank test between dose groups, p = 0.633), although the study was not powered to detect a difference between doses.

The primary efficacy endpoint was also evaluated for the per-protocol population, and the results were consistent with those for the EFF population; the Kaplan–Meier-estimated exit rate was 10.9% [95% CI 5.6–20.5%] for ESL 1600 mg and 12.8% [6.0–26.3%] for ESL 1200 mg. Again, although the study was not powered to detect a difference between ESL doses, the exit rates were similar for both dose groups (p = 0.723).

#### Secondary analyses of the primary endpoint

In a secondary analysis of the primary efficacy endpoint (without reassignment of ‘non-exit withdrawals’ as exits), the Kaplan–Meier-estimated exit rates were 12.8% [95% CI 7.5–21.5%] and 13.6% [6.7–26.5%] for ESL 1600 and 1200 mg, respectively. The difference in exit rates between ESL dose groups was not statistically significant (p = 0.861).

**Figure 5:**
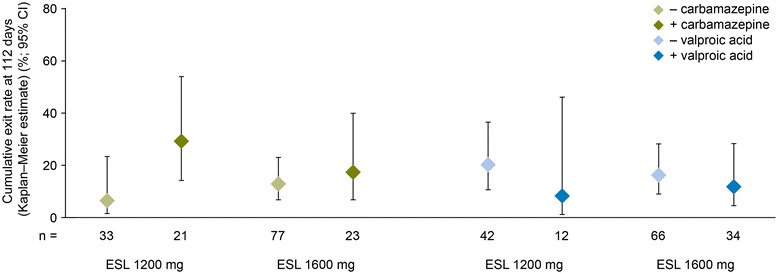
**Kaplan–Meier estimates of exit rate at 112 days with/without carbamazepine and valproic acid (EFF population).** EFF = efficacy; CI = confidence interval; ESL = eslicarbazepine acetate.

A total of 44 patients (EFF population) were taking CBZ during the baseline period (ESL 1200 mg, n = 21; 1600 mg, n = 23), while 46 patients were taking valproic acid (ESL 1200 mg, n = 12; 1600 mg, n = 34). The Kaplan–Meier-estimated exit rates for patients taking CBZ at baseline (ESL 1200 mg, 29.3% [95% CI 14.3–54.0%]; ESL 1600 mg, 17.4% [6.9–39.9%]) were numerically higher than for those not taking CBZ (ESL 1200 mg, 6.5% [1.7–23.4%]; ESL 1600 mg, 12.8% [6.9–23.1%]; Figure 5). In contrast, the Kaplan–Meier-estimated exit rates for patients taking valproic acid at baseline (ESL 1200 mg, 8.3% [1.2–46.1%]; ESL 1600 mg, 11.8% [4.6–28.4%]) were lower than for those not taking valproic acid (ESL 1200 mg, 20.3% [10.7–36.6%]; ESL 1600 mg, 16.3% [9.1–28.2%]; Figure 5). Irrespective of CBZ and valproic acid use during the baseline period, the UCLs for the Kaplan–Meier-estimated exit rates for both ESL dose groups were below the 65.3% threshold (calculated from the historical controls).

The effects of covariates were assessed using a Cox proportional hazards regression model. The adjusted exit rates changed minimally when adjusted for baseline seizure frequency, age, number of overall AEDs, and number of background AEDs used at baseline.

#### Secondary endpoints

Four patients (7.4% [95% CI 2.1–17.9%]) on ESL 1200 mg and 10 (10% [4.9–17.6%]) on ESL 1600 mg were seizure free during the 10-week monotherapy period, while seizure-free rates during the last 4 weeks of monotherapy were 16.7% [95% CI 7.9–29.3%] for ESL 1200 mg and 17% [10.2–25.8%] for ESL 1600 mg. Completion rates for the 18-week double-blind treatment period were 75.9% [95% CI 62.4–86.5%] for ESL 1200 mg and 80% [70.8–87.3%] for ESL 1600 mg, and for the 10-week monotherapy period were 85.4% [95% CI 72.2–93.9%] for ESL 1200 mg and 90.9% [82.9–96.0%] for ESL 1600 mg.

Seizure frequency was calculated as the standardized seizure frequency per 28 days. The median reduction in standardized seizure frequency between the baseline period and the 18-week double-blind period was 36.1% for ESL 1200 mg and 47.5% for ESL 1600 mg; >80% of patients treated with ESL had fewer seizures during the 18-week double-blind period, compared with the baseline period. The difference between the ESL dose groups was not significant (p = 0.563). The median reduction in standardized seizure frequency between the baseline period and the monotherapy period was 45.7% for ESL 1200 mg and 52.1% for ESL 1600 mg (Table 4). Responder rates (% patients with ≥50% reduction in seizure frequency versus baseline) for ESL 1200 and 1600 mg were 35.2% [95% CI 22.7–49.4%] and 46.0% [36.0–56.3%] for the double-blind period and 38.9% [29.5–58.8%] and 46.0% [41.4–63.0%] for the monotherapy period, respectively (Table 4).

**Table 4:** **Percentage change from baseline in standardized seizure frequency during the monotherapy and double-blind periods, and responder rate by study period (EFF population)**

**Study period**	**ESL 1200 mg (n = 54)**	**ESL 1600 mg (n = 100)**
% change in SSF from baseline for the 10-week monotherapy period		
Mean ± SD	−42.3 ± 42.48	−39.2 ± 57.93
Median	−45.7	−52.1
% change in SFF from baseline for the 18-week double-blind period		
Mean ± SD	−33.0 ± 43.33	−37.3 ± 46.26
Median	−36.1	−47.5
Responder rate		
Titration period; n (%) [95% CI]	16 (29.6%) [18.0–43.6%]	37 (37.0%) [27.6–47.2%]
AED conversion period; n (%) [95% CI]	16 (29.6%) [18.0–43.6%]	39 (39.0%) [29.4–49.3%]
Monotherapy period; n (%) [95% CI]	21 (38.9%) [29.5–58.8%]	46 (46.0%) [41.4–63.0%]
Double-blind period; n (%) [95% CI]	19 (35.2%) [22.7–49.4%]	46 (46.0%) [36.0–56.3%]

Responder rate was defined as percentage of patients with a ≥50% reduction in seizure frequency from baseline. Percentages of responders and 95% CIs are based on the number of patients with post-baseline seizure data.

*EFF* = efficacy; *ESL* = eslicarbazepine acetate; *SSF* = standardized seizure frequency (seizure frequency is standardized to a 28-day frequency); *SD* = standard deviation; *CI* = confidence interval; *AED* = antiepileptic drug.

With the exception of nine patients with simple partial seizures without motor symptoms, patients with all other seizure types had reductions in standardized seizure frequency of between 39% and 80% (between the baseline and monotherapy periods; Table 5). Responder rates during the monotherapy period were 41–73% for all seizure types (again, except for the patients with simple partial seizures without motor symptoms).

**Table 5:** **Percentage change from baseline in standardized seizure frequency, and responder rate by seizure type during the monotherapy period (EFF population)**

	**n**		**Median change from baseline (%)**		**Responder rate (%)** ^*^	
	**ESL 1200 mg**	**ESL 1600 mg**	**ESL 1200 mg**	**ESL 1600 mg**	**ESL 1200 mg**	**ESL 1600 mg**
Simple partial without motor	2	7	22.4	−100.0	0	85.7
Simple partial with motor	17	34	−38.5	−52.0	41.2	52.9
Complex partial	30	53	−70.9	−49.8	66.7	49.1
Partial evolving to secondary generalized	16	33	−51.3	−79.7	50.0	72.7

^*^Patients with ≥50% reduction; calculated as percentage of patients in ESL dose group.

*EFF* = efficacy; *ESL* = eslicarbazepine acetate.

Treatment with ESL was associated with an increase in total QOLIE-31 scores between baseline and the end of the monotherapy period (by 4.0 ± 11.5 points with ESL 1200 mg, and by 4.7 ± 13.7 points with ESL 1600 mg), and a reduction in total MADRS scores (by 1.6 ± 4.5 points with ESL 1600 mg; unchanged [±6.5] with ESL 1200 mg). More marked reductions were apparent in patients with MADRS scores ≥14 at baseline (4.1 ± 7.6 points with ESL 1600 mg; 6.1 ± 6.7 points with ESL 1200 mg).

### Safety

Overall, 116 patients (67%) reported ≥1 TEAE during the study (60% in the ESL 1200 mg group and 71% in the ESL 1600 mg group). Among patients who had ≥1 TEAE (n = 116), 78% reported a TEAE that was considered potentially related to the study drug (less frequent for ESL 1200 mg [66%] than ESL 1600 mg [83%]). The most commonly reported TEAEs were headache (25% of patients), dizziness (17%), nasopharyngitis (8%), nausea (8%), somnolence (7%), fatigue (6%) and back pain (5%) (Table 6). A greater proportion of patients reported TEAEs during the titration period (42%) than the AED conversion period (37%) and the monotherapy period (38%). The most common TEAEs reported during the monotherapy period (in ≥2% of patients) were headache (12%), back pain and nausea (both 4%), influenza, nasopharyngitis, complex partial seizures, and dizziness (2% each).

**Table 6:** **TEAEs affecting ≥5% of patients in any ESL dose group (ITT population, all periods)**

**TEAE; n (%)**	**ESL 1200 mg (n = 58)**	**ESL 1600 mg (n = 114)**	**Total (n = 172)**
Headache	11 (19.0)	32 (28.1)	43 (25.0)
Dizziness	6 (10.3)	24 (21.1)	30 (17.4)
Nasopharyngitis	4 (6.9)	9 (7.9)	13 (7.6)
Nausea	6 (10.3)	7 (6.1)	13 (7.6)
Somnolence	2 (3.4)	10 (8.8)	12 (7.0)
Fatigue	4 (6.9)	6 (5.3)	10 (5.8)
Back pain	3 (5.2)	6 (5.3)	9 (5.2)
Insomnia	4 (6.9)	3 (2.6)	7 (4.1)
Complex partial seizures	1 (1.7)	6 (5.3)	7 (4.1)
Influenza	3 (5.2)	1 (0.9)	4 (2.3)
Anxiety	3 (5.2)	1 (0.9)	4 (2.3)

*TEAE* = treatment-emergent adverse event; *ESL* = eslicarbazepine acetate; *ITT* = intention-to-treat.

Most TEAEs reported during the study were mild or moderate in severity. Overall, 64 patients (37%) reported ≥1 TEAE of mild intensity, and 48 (28%) reported ≥1 TEAE of moderate intensity. During the AED conversion and monotherapy periods, severe TEAEs were reported by four patients (3.5% of the ESL 1600 mg group, 2.3% of the total). Eight patients on ESL 1600 mg (7.0%) and one on ESL 1200 mg (1.7%) reported a treatment-emergent serious AE; none were fatal. The single serious AE reported for ESL 1200 mg was atrial flutter (which occurred prior to dosing with ESL), while the ten serious AEs reported for ESL 1600 mg (by eight patients) were: ankle fracture; post-concussion syndrome; tibia fracture; hyponatremia; complex partial seizures; partial seizures with secondary generalization; syncope; spontaneous abortion; drug rash with eosinophilia and systemic symptoms (DRESS); and pruritic rash (1 event in 1 patient each). Most serious AEs occurred during the AED conversion period (2.3% of all patients). There were no deaths during the 18-week double-blind period. One patient died due to a convulsion during the baseline period, before receiving study drug.

Overall, 16 patients (9%) discontinued the study due to a TEAE (3% on ESL 1200 mg versus 12% on ESL 1600 mg). Thirteen patients (8%) discontinued due to TEAE that was potentially related to ESL. The most common TEAEs leading to study discontinuation were complex partial seizures (2.3%) and pruritic rash (1.2%). More TEAEs leading to discontinuation were reported during the titration period (5%) than the AED conversion period (2%) and the monotherapy period (2%). The frequency of dose reductions due to TEAEs was similar between the ESL 1200 mg group (5%) and the ESL 1600 mg group (4%); dose reductions occurred only during the AED conversion period.

Most clinical laboratory parameters were comparable between treatment groups. Decreased plasma sodium (125–135 mEq/L) was noted in 75% and 58% of patients on ESL 1600 and 1200 mg, respectively; no patients had plasma sodium <125 mEq/L. 15% of patients had a reduction in plasma sodium ≥10 mEq/L from baseline at some time during the 18-week double-blind period. There were no clinically relevant changes in vital signs, no orthostatic effects, no significant abnormalities in physical and neurological examinations in either treatment group, and no clinically significant electrocardiogram findings. Suicidality (assessed using the Columbia Suicide Severity Rating Scale [C-SSRS] questionnaire) was reported post baseline in 3% of the ESL 1600 mg group and 3% of the ESL 1200 mg group. Suicidality was not reported as a TEAE.

## Discussion

The current study met its primary efficacy endpoint (the proportion of patients who exited the study on meeting at least one exit criterion, e.g., due to poor seizure control) by demonstrating that the exit rates for patients who converted to ESL monotherapy (both dose levels) were <16% and were statistically lower than the exit rates in the pseudo-placebo arms of the historical control trials. Thus, the efficacy of ESL monotherapy (1600 and 1200 mg QD) for seizure control was demonstrated to be superior to the historical control.

Exit rates for patients who took CBZ during the baseline period were numerically higher than for those not taking CBZ. In their analysis of eight study cohorts, French et al. [17] found that withdrawal from CBZ did not significantly increase the likelihood of exiting a trial, but increased the hazard rate of exiting by 8.0% [95% CI –19.4, 35.4%]. In the current trial, both patient subgroups (CBZ users and non-users) had exit rates that were significantly different from the 65.3% threshold. It should be noted, however, that this threshold was computed for the total historical group, including both users and non-users of CBZ [17]. In the current trial, exit rates for patients receiving valproic acid at baseline were numerically lower than for patients not receiving valproic acid as a baseline AED.

There was a reduction in standardized seizure frequency (in both ESL dose groups) from baseline across different seizure types; due to the small sample size for patients with simple partial seizures without motor symptoms, it was not possible to draw conclusions about the efficacy of ESL for this type of seizure.

The improvements observed with ESL in several of the secondary efficacy endpoints (proportion of seizure-free patients during monotherapy; reduction in standardized seizure frequency; responder rate) are consistent with the suggestion that ESL monotherapy is a potential treatment option for patients with POS.

A 4-point improvement in QOLIE-31 score occurred in both ESL treatment groups, suggesting that patients perceived some improvement in their quality of life. However, according to Borghs et al. [23], the minimum clinically relevant improvement in QOLIE-31 score is a 5-point improvement, which indicates that the improvement in QOLIE-31 that occurred during the current trial may not be clinically relevant. A longer period of observation may be required to identify changes in quality of life. In contrast, the improvement in depressive symptoms during treatment with ESL 1600 mg (as indicated by a 1.6-point reduction in MADRS score) does appear to be clinically relevant, being comparable to the minimal clinically important difference (a 1.6–1.9 point improvement) described by Duru and Fantino [24]. In patients with at least mild depressive symptoms at baseline (MADRS scores ≥14), clinically important differences in MADRS scores were seen in both ESL treatment groups.

The majority of patients (>75%) completed the 10-week monotherapy period and the total (18-week) study period, indicating that ESL monotherapy was well tolerated. Compared with the safety profile of ESL determined in the earlier adjunctive trials, no new safety issues were raised during the current trial, and the AEs observed were consistent with the previously reported safety profile of ESL [10]. The most commonly reported TEAEs were headache and dizziness, of mild to moderate severity. TEAEs were reported more frequently by patients on ESL 1600 mg than by those on ESL 1200 mg. Severe TEAEs were only reported for the ESL 1600 mg dose group, during the AED conversion and monotherapy periods. Serious AEs were mostly reported for the 1600 mg ESL group; the incidence of serious AEs was low during the monotherapy period. The TEAEs that most often led to study discontinuation were complex partial seizures and pruritic rash (incidence <2.5% each). There were no treatment-emergent deaths.

This study adopted the “historical control withdrawal to monotherapy design” described by French et al. [17] to evaluate the safety and efficacy of ESL monotherapy in adults with POS not well controlled by one or two AEDs. The use of a historical control is a potential limitation of this study. The historical control withdrawal to monotherapy study design is regarded as ethical and reliable for investigating the efficacy of AEDs in the monotherapy setting [17]. The fact that the design, patient population, evaluation criteria, and analysis plan of the current study are comparable to those of the historical control studies means that it is justifiable to compare the respective exit rates [25]. One factor that may have influenced the results is that the proportion of patients taking CBZ at baseline in this study was lower (28.6% overall) than in the historical control trials (see Additional file 1: Table S2). As patients who have converted from CBZ-based therapy have been shown to exhibit higher exit rates than others, this might contribute to the differences in overall exit rates between the current trial and the historical control. The use of historical controls could have influenced the study outcome, because all patients would have been aware that they were receiving active treatment. However, this was also the case for the historical comparator trials (which used a “pseudo-placebo” comparator). Moreover, the influence of this effect should be minimized by the blinded randomization to the two dose arms used in this study. Previous studies have indicated that patients can experience a ‘honeymoon period’ when beginning with a new AED; patients may show initial improvement, but may become resistant on exposure to prolonged treatment [26,27]. This is often due to pharmacokinetic or pharmacodynamic tolerance [28]. The duration of the current trial exceeded the estimated ‘honeymoon period’ (51–82 days with levetiracetam [27]), and was therefore of sufficient duration to detect any similar tolerance with ESL. A long-term open-label extension study is underway, with the objective of evaluating whether the efficacy and safety of ESL monotherapy is maintained during long-term use.

Other recent epilepsy studies have used this approach to investigate the use of AEDs as monotherapy (levetiracetam XR 2000 mg QD [29]; lamotrigine XR 300 and 250 mg QD [25]; lacosamide 300 and 400 mg/day [30]; pregabalin 600 mg/day [31]). All these studies recruited patients from a mix of US and ex-US populations, as did the current study, whereas the eight studies comprising the historical control [17] were recruited exclusively from North America. It is unclear whether this had an important influence on the study results. It should also be noted that there are subtle but potentially important differences among these trials. The current study used a stringent definition of the primary endpoint. First, once the withdrawal rate had exceeded 10%, further withdrawals were reassigned as seizure exits by random sampling. Second, information from the seizure diaries was used to evaluate seizure rate, and patients whose rate at least doubled between baseline and the 16-week study period were also reassigned as seizure exits (if the investigator had not already done so). This was not done in the levetiracetam XR, lamotrigine XR, lacosamide or pregabalin trials. The lamotrigine XR trial excluded patients using CBZ as a baseline medication, when CBZ is known to affect the exit rate in studies of this design. Therefore caution must be used when comparing exit rates among these studies. Two conversion to monotherapy trials of brivaracetam (ClinicalTrials.gov identifiers: NCT00698581 and NCT00699283) using historical controls were terminated after an interim analysis demonstrated trial futility.

A second trial (study 045) examining the efficacy and safety of ESL has been performed in a 100% North American population [21], using an identical protocol. The efficacy of ESL monotherapy for seizure control in study 045 was also found to be superior to historical controls. However, the Kaplan–Meier-estimated exit rates in study 045 were higher (1600 mg: 28.7% [95% CI 21.2–38.1%]; 1200 mg: 44.4% [32.5–58.3%]) than the rates observed in this study, potentially due to the difference in study populations.

## Conclusions

The results of this phase III study demonstrate that ESL monotherapy, following conversion from other adjunctive AEDs, was effective based on comparison with a historical control. Additionally, during ESL monotherapy a substantial fraction of patients experienced a reduction in seizure frequency compared with baseline. The relatively high completion rate and the side effect profile of ESL at doses of 1200 and 1600 mg QD indicate that ESL was efficacious and well tolerated when used as monotherapy.

## Additional file

Additional file 1: Table S1. Baseline AEDs (EFF population). **Table S2.** Demographic and clinical characteristics of patients in the trials comprising the historical control. **Appendix S1.** Statistical analyses. **Appendix S2.** The Study 046 team.

## Abbreviations

AE: Adverse event
AED: Antiepileptic drug
CBZ: Carbamazepine
CI: Confidence interval
C-SSRS: Columbia Suicide Severity Rating Scale
DRESS: Drug rash with eosinophilia and systemic symptoms
EFF: Efficacy
ESL: Eslicarbazepine acetate
ITT: Intention-to-treat
MADRS: Montgomery–Asberg Depression Rating Scale
MedDRA: Medical Dictionary for Regulatory Activities
OXC: Oxcarbazepine
POS: Partial-onset seizures
QD: Once daily
QOLIE-31: Quality of Life in Epilepsy – 31 item inventory
TEAE: Treatment-emergent adverse event
UCL: Upper 95% confidence limit
VGSC: Voltage-gated sodium channel
