# Supplementary material for: Efficacy and safety of conversion to monotherapy with eslicarbazepine acetate in adults with uncontrolled partial-onset seizures: a historical-control phase III study
Source: BMC Neurol. 2015 Mar 28;15:46. doi: 10.1186/s12883-015-0305-5 (PMC4397697; doi:10.1186/s12883-015-0305-5)
Supplement: Additional file 1: Table S1. — Baseline AEDs (EFF population). Table S2. Demographic and clinical characteristics of patients in the trials comprising the historical control. Appendix S1. Statistical analyses. Appendix S2. The Study 046 team. [file 12883_2015_305_MOESM1_ESM.docx]

# additional file 1

# ONLINE SUPPORTING INFORMATION

## Table S1 Baseline AEDs (EFF population)

| **Baseline AED, n (%)** | **ESL 1200 mg**  **(n = 54)** | **ESL 1600 mg QD**  **(n = 100)** | **Total ESL**  **(n = 154)** |
| --- | --- | --- | --- |
| Any AED | 54 (100) | 100 (100) | 154 (100) |
| Valproic acid | 12 (22.2) | 34 (34.0) | 46 (29.9) |
| Carbamazepine | 21 (38.9) | 23 (23.0) | 44 (28.6) |
| Levetiracetam | 10 (18.5) | 19 (19.0) | 29 (18.8) |
| Lamotrigine | 10 (18.5) | 16 (16.0) | 26 (16.9) |
| Topiramate | 4 (7.4) | 17 (17.0) | 21 (13.6) |
| Phenytoin | 4 (7.4) | 8 (8.0) | 12 (7.8) |
| Oxcarbazepine | 2 (3.7) | 8 (8.0) | 10 (6.5) |
| Pregabalin | 0 | 6 (6.0) | 6 (3.9) |
| Lacosamide | 1 (1.9) | 4 (4.0) | 5 (3.2) |
| Gabapentin | 2 (3.7) | 2 (2.0) | 4 (2.6) |
| Zonisamide | 2 (3.7) | 2 (2.0) | 4 (2.6) |
| Sultiame | 1 (1.9) | 0 | 1 (0.6) |

AED = antiepileptic drug; EFF = efficacy; ESL = eslicarbazepine acetate; QD = once daily.

**Table S2 Demographic and clinical characteristics of patients in the trials comprising the historical control**

| **Characteristic** | **Beydoun et al, 1997** | **Gilliam et al, 1998** | **Sachdeo et al, 1997** | **Unpublished** | **Sachdeo et al, 2001** | **Beydoun et al, 2000** | **Sachdeo et al, 1992** | **Faught et al, 1993** |
| --- | --- | --- | --- | --- | --- | --- | --- | --- |
| Age, years; mean (range) | 35 (14–63) | 36 (14–71) | 35 (NA) | NA | 35 (18–53) | 36 (11–66) | 38 (18–62) | 35 (17–67) |
| Male, % | 55.3 | 40 | 38 | NA | 53 | 41 | 25 | 36.3 |
| Race |  |  |  |  |  |  |  |  |
| White, % | NA | 69 | 83 | NA | 87 | NA | NA | 85.5 |
| Black, % | NA | 14 | 4 | NA | 0 | NA | NA | 9.1 |
| Other, % | NA | 17 | 13 | NA | 13 | NA | NA | 5.5 |
| Countries | US, Canada | US | US | US | US | US | US | US |
| 28-day seizure frequency at baseline, median/mean | 6.5/10.1 | 10.0/NA | 9.5/65.0 | NA | 5.5/NA | 6.5/NA | NA/15.9 | NA/21.3 |
| Minimum seizure frequency at baseline | 4/8wks | 4/4wks | 4 /4wks | NA | 2/4wks | 2/4wks | 8/8wks | 8/8wks |
| AEDs used at baseline |  |  |  |  |  |  |  |  |
| Carbamazepine, % | 64 | 58 | 63 | NA | NA | 46 | 59 | NA |
| Valproic acid, % | 28 | 0 | 13 | NA | NA | 13 | 23 | NA |
| Number of AEDs used during baseline | 1–2 | 1 | 1–2 | NA | 1 | 1–2 | 1–2 | 1–2 |
| One, %* | 66 | 100 | 83 | NA | 100 | NA | 68 | NA |
| Two, % | 34 | 0 | 17 | NA | 0 | NA | 32 | NA |
| Epilepsy duration, years; median (range) | 21 (<1–45) | NA | 21 (mean) | NA | NA | NA | NA | NA |
| Patients with complex partial seizures, % | 95 | 89 | 83 | NA | 87 | NA | NA | NA |
| Duration of baseline period (weeks) | 8 | 8 | 8 | NA | 8 | 8 | 8 | 8 |
| Duration of double-blind period (weeks) | 26 | 20 | 16 | NA | 18 | 18 | 16 | 16 |
| Duration of withdrawal period (weeks) | 10 | 8 | 5 | NA | 6 | 6 | 4 | 4 |
| Duration of monotherapy period (weeks) | 16 | 12 | 11 | NA | 12 | 12 | 12 | 12 |

*Calculated values.

AED = antiepileptic drug; NA = not available/not applicable; US = United States.

**References**

1. Beydoun A, Fischer J, Labar DR, Harden C, Cantrell D, Uthman BM, Sackellares JC, Abou-Khalil B, Ramsay RE, Hayes A, Greiner M, Garofalo E, Pierce M: Gabapentin monotherapy: II. A 26-week, double-blind, dose-controlled, multicenter study of conversion from polytherapy in outpatients with refractory complex partial or secondarily generalized seizures. The US Gabapentin Study Group 82/83. *Neurology* 1997, **49**:746–752.

2. Gilliam F, Vazquez B, Sackellares JC, Chang GY, Messenheimer J, Nyberg J, Risner ME, Rudd GD. An active-control trial of lamotrigine monotherapy for partial seizures. *Neurology* 1998, **51**:1018–1025.

3. Sachdeo RC, Reife RA, Lim P, Pledger G: Topiramate monotherapy for partial onset seizures. *Epilepsia* 1997, **38**:294–300.

4. Sachdeo R, Beydoun A, Schachter S, Vazquez B, Schaul N, Mesenbrink P, Kramer L, D’Souza J: Oxcarbazepine (Trileptal) as monotherapy in patients with partial seizures. *Neurology* 2001, **57**:864–871.

5. Beydoun A, Sachdeo RC, Rosenfeld WE, Krauss GL, Sessler N, Mesenbrink P, Kramer L, D’Souza J: Oxcarbazepine monotherapy for partial-onset seizures: a multicenter, double-blind, clinical trial. *Neurology* 2000, **54**:2245–2251.

6. Sachdeo R, Kramer LD, Rosenberg A, Sachdeo S: Felbamate monotherapy: controlled trial in patients with partial onset seizures. *Ann Neurol* 1992, **32**:386–392.

7. Faught E, Sachdeo RC, Remler MP, Chayasirisobhon S, Iragui-Madoz VJ, Ramsay RE, Sutula TP, Kanner A, Harner RN, Kuzniecky R, Kramer LO, Kamin M, Rosenberg I: Felbamate monotherapy for partial-onset seizures: an active-control trial. *Neurology* 1993, **43**:688–692.

**Appendix S1 Statistical analyses**

## Key secondary efficacy analysis

The number and proportion (%) of subjects who were seizure-free during 10-week double-blind monotherapy treatment (along with the 95% confidence interval [CI]) was calculated (using binomial methods) for each dose group. The proportion was calculated as the number of seizure-free subjects divided by the total number of subjects in the efficacy (EFF) population for each dose group.

Seizure-free subjects for the analysis of this endpoint were subjects who met all of the following criteria:

- Had at least one seizure assessment during the monotherapy period (Visits 6 through 9).
- Did not have any seizures during the 10-week monotherapy period (Visits 6 through 9 [subjects who were seizure-free but discontinued during the 10-week monotherapy period were not considered seizure-free for this analysis]).

## Other secondary efficacy analyses

### Seizure-free during last 4 weeks

Seizure-free subjects for the analysis of this endpoint were subjects who met both of the following criteria:

- Had at least one seizure assessment from Visit 8 through Visit 9.
- Did not have any seizures from start of Visit 8 through Visit 9 (subjects who were seizure-free but discontinued during the last 4 weeks of the monotherapy period were not considered seizure-free for this analysis).

The proportion was calculated as the number of seizure-free subjects divided by the total number of subjects in the EFF population for each dose group. The number and proportion (%) of subjects seizure-free during the last four weeks (Visit 8 through Visit 9) of the 10-week double-blind eslicarbazepine acetate (ESL) monotherapy period was computed, along with the 95% CI (using binomial methods) for each dose group.

### Study completion rate

The study completion rate was defined as the proportion (%) of subjects in the EFF population who completed the entire 18 weeks of ESL double-blind treatment (Visit 2 through Visit 9). The study completion rate was computed along with the 95% CI (using binomial methods) for each dose group.

### Monotherapy completion rate

Completion rate during the 10-weeks of double-blind ESL monotherapy (Visit 6 through Visit 9) was defined as the proportion (%) of subjects who entered the double-blind ESL monotherapy period (Visit 6) and who completed 10-weeks of double-blind ESL monotherapy treatment (Visit 9). Subjects who entered the double-blind ESL monotherapy period are defined as subjects who terminated all baseline antiepileptic drugs (AEDs) while taking study drug (ESL) (Visit 6). The monotherapy completion rate was computed along with the 95% CI (using binomial methods) for each dose group.

### Standardized seizure frequency by period and by seizure type

Standardized seizure frequency was calculated using seizure frequency standardized to a frequency per 4 weeks (28 days). It was calculated for four periods: titration (Visit 2 to Visit 4); AED taper/conversion (Visit 4 to Visit 6); ESL monotherapy (Visit 6 to Visit 9); and double-blind (Visit 2 to Visit 9).

The standardized seizure frequency for any given period (including baseline) was calculated as:

$$\frac{\begin{aligned} \text{Total number of seizures reported in the diary } \\ \text{during the interval of interest} \end{aligned}}{\begin{aligned} \text{Number of days on study for the}\text{ } \\ \text{ interval of interest} \end{aligned}}*28 days$$

Standardized seizure frequency was summarized descriptively by period. In addition, the double-blind period and monotherapy period seizure frequency (log transformed) were analyzed by an analysis of covariance (ANCOVA) model with baseline seizure frequency as covariate and dose group as a fixed effect. Least-squares (LS) means of seizure frequency were estimated for each dose group, along with 95% CIs. Standard errors were calculated using the delta method (standard error [SE] = exp[LS mean value]* SE of the LS mean). A constant (c = 0.333) was added to the standardized seizure frequencies prior to log transformation. Model estimates were back transformed to original scale by the following equation (exp[LSMEANS] – 0.333). The LS means differences in standardized seizure frequencies between the two dose groups along with the 95% CIs were presented. This analysis was also presented by seizure type.

### Relative (%) change in standardized seizure frequency by period and by seizure type

The relative (%) change in seizure frequency was evaluated for four periods: titration (Visit 2 to Visit 4); AED taper/conversion (Visit 4 to Visit 6); ESL monotherapy (Visit 6 to Visit 9); and double-blind (Visit 2 to Visit 9). It was calculated as:

$$\frac{\begin{aligned} \text{Standardized seizure frequency for the interval of interest -} \\ \text{Standardized baseline seizure frequency} \end{aligned}}{\text{Standardized baseline seizure frequency}}*100$$

The relative (%) change in standardized seizure frequency was summarized descriptively by period. In addition, the double-blind period and monotherapy period relative (%) change in seizure frequency were analyzed by an ANCOVA model with baseline seizure frequency as covariate and dose group as a fixed effect. Finally, the number and percentage of subjects in the following categories were presented for each period by dose group: 100% decrease; ≥75% to <100% decrease; ≥50% to <75% decrease; 0% to <50% decrease; >0% to <25% increase; ≤25% increase. This analysis was also presented by seizure type.

### Responder rate

Responder rate was defined as percent of subjects with a ≥ 50% reduction of seizure frequency from baseline and was presented along with 95% CIs (using binomial methods) by period for both dose groups. This analysis was performed for four periods: the titration (Visit 2 to Visit 4); AED taper/conversion (Visit 4 to Visit 6); ESL monotherapy (Visit 6 to Visit 9); and double-blind (Visit 2 to Visit 9). Seizure reduction was based on standardized seizure frequency per 4 weeks. The proportion (%) of responders was calculated as the number of responders divided by the total number of subjects in the EFF population who entered the period of interest.

### Proportion of subjects reaching each exit criteria

The proportion (%) of subjects reaching each of the five exit criteria during the 112-day period was calculated for each dose group along with 95% CIs using binomial methods. A subject reaching more than one exit criterion was counted based on the first occurrence. The proportion (%) was calculated as the number of subjects reaching each of the five exit events divided by the total number of subjects in the EFF population. For exit criteria 3 and 4, both the Investigator’s determination of the exit criteria as well as the programmatic derivation based on seizure diary data was presented. The proportion of subjects meeting the exit criteria, along with 95% CIs were calculated using binomial methods for each treatment group.

### 31-item Quality of Life in Epilepsy (QOLIE-31) change from baseline

Observed values and change from baseline in the seven individual items, overall score, and global assessment to each post-baseline assessment in QOLIE-31 was summarized by dose group using descriptive statistics.

### Montgomery–Åsberg depression scale (MADRS) change from baseline

The total MADRS score and the ten individual items at baseline and at Weeks 8 (Visit 6) and 18 (Visit 9) was summarized descriptively by dose group. The change from baseline in the total score was summarized descriptively along with the LS means and corresponding confidence limits from an ANCOVA model including baseline total score as a covariate and dose group as a fixed effect. The same analyses were performed for subjects with a MADRS total score of ≥ 14 at baseline.

## Safety analysis

All safety variables were analyzed descriptively for the intent-to-treat (ITT) population. Safety variables included adverse events (AEs), clinical laboratory tests (serum chemistry, hematology, and urinalysis), coagulation test, thyroid panel, lipid panel, bone turnover markers, blood sodium levels, electrocardiograms (ECGs), vital sign measurements, orthostatic effects, physical examination, bodyweight, concomitant medication use, and Columbia Suicide Severity Rating Scale (C-SSRS).

### AEs

All AEs were coded using the Medical Dictionary for Regulatory Activities Version 13.1 and monitored from Visits 1 through 10.

Treatment-emergent AEs (TEAEs) were defined as:

- AEs that occurred on or after the first dose of study drug.
- AEs with a missing start date and a stop date on or after the first dose of study drug.
- AEs with both a missing start and stop date.

A summary of the pre-randomization (screening and baseline) period adverse events (non-TEAEs) was presented for the overall ITT population. If a subject has more than one occurrence of an event in the same System Organ Class (SOC) and/or Preferred Term (PT), the subject was counted only once within that SOC and/or PT for the subject incidence summaries. The following summaries were created for all TEAEs and by study period for each dose group and overall:

- TEAEs, including subject incidence and number of events.
- TEAEs by severity (mild, moderate, severe).
- TEAEs by relationship to treatment (unrelated or potentially related).
- Serious TEAEs.
- TEAEs leading to discontinuation of treatment.

In addition, the summary of TEAEs was presented by week of the double-blind period for each treatment group. Study periods were defined as:

- Titration period (Visit 2 to the day before Visit 4).
- AED taper/conversion period (Visit 4 to the day before Visit 6).
- ESL monotherapy period (Visit 6 [termination of all baseline AEDs] to Visit 9).
- Taper period (Day after Visit 9 to Visit 10).

### Severity

TEAEs were summarized by SOC, PT, and severity. If a subject had more than one TEAE within a PT and/or SOC, the TEAE was counted at its maximum severity within each SOC and PT. The number and percentage of subjects with AEs by severity were summarized overall and by study period.

### AEs leading to dose reduction or discontinuation

The number and percentage of subjects with dose reductions due to AEs were summarized for each dose group for the overall study and by study phase. Descriptive statistics of the time (days) to TEAEs leading to dose reduction was also included. The number and percentage of subjects with AEs leading to study discontinuation were summarized overall and by study period. Listings of AEs leading to discontinuation were presented.

### AEs of special interest

All unique PTs were reviewed prior to database lock and unblinding to identify rash-related AEs. Listings of rash-related AEs and adverse events of special interest were presented.

### Clinical laboratory analysis

Descriptive statistics for test results and change from baseline values were displayed at each visit for each continuous laboratory parameter. For laboratory parameters with categorical outcomes, the number and percentage of subjects with each outcome were presented for each dose group and overall. The normal reference ranges for laboratory tests were used to determine whether the laboratory test value was below, within, or above the normal range. Shifts from baseline to each visit were produced to show the percentage of subjects with laboratory test values below, within, and above the normal range. The percentage was based on the number of subjects with a baseline result and at least one post-baseline result for each parameter. The listings for laboratory parameters flagged values outside of the reference range. These were also listed separately. The number and percentage of subjects with normal baseline laboratory levels reaching the following potentially clinically significant (PCS) laboratory values during the double-blind treatment period were summarized for each dose group and overall. A listing of all PCS laboratory parameters presented all values for that subject and parameter, flagging the values meeting the PCS criteria.

### Blood sodium levels

In addition to continuous summaries of blood sodium levels, the number and percentage of subjects with normal baseline sodium levels reaching the following post-baseline blood sodium level were identified and summarized by time-point and overall post-baseline for each dose group and overall:

- ≤135 mEq/L but > 130 mEq/L.
- ≤130 mEq/L but > 125 mEq/L.
- ≤125 mEq/L.

### ECG evaluations

Descriptive statistics for each parameter and change from baseline values were displayed at each visit for each ECG parameter by dose group and overall. Change from baseline in the following ECG parameters was calculated: ventricular HR, QT interval, PR interval, QRS durations, RR interval, and the QTc intervals corrected by Bazett (QTcB) and Fridericia (QTcF). QTcB was calculated as QT interval*(square root of [HR/60]). QTcF was calculated as QT interval*(cube root of [HR/60]).

Additionally, the number and percentage of subjects with QTc-F values in the following categories during the double-blind treatment period were identified and summarized for each dose group and overall:

- QTc-F >500 ms at any post-baseline time point not present at baseline.
- QTc-F >480 ms at any post-baseline time point not present at baseline.
- QTc-F >450 ms at any post-baseline time point not present at baseline.
- Change from baseline in QTc-F ≥60 ms for at least one post-baseline measurement.
- Change from baseline in QTc-F ≥30 ms for at least one post-baseline measurement, but <60 ms for all post-baseline measurements.

This categorical analysis was also performed for QTc-B.

The number and percentage of subjects with ECG abnormalities in the categories of overall, rhythm, conduction, morphology, myocardial infarction and the presence of ST, T, and U wave abnormalities during the double-blind treatment period were presented for each dose group and overall.

### Vital signs

The number and percentage of subjects meeting the Sponsor-defined vital sign PCS criteria (including increase of body weight ≥7%) during the double-blind treatment period were summarized by dose group and overall. A vital sign value was considered PCS low and was flagged if it was below the specified low limit and it decreased from baseline more than the specified decrease from baseline limit. A vital sign value was considered PCS high and was flagged if it was higher than the specified high limit and it increased from baseline more than the specified increase from baseline limit. A data listing of vital signs meeting the PCS criteria will be provided with PCS flags.

### Orthostatic effects

Orthostatic effects were evaluated as part of the vital signs analysis. Orthostatic hypotension is defined as a decrease of ≥20 mmHg in standing systolic or ≥ 10 mmHg in standing diastolic blood pressure, as compared to the supine position. Orthostatic tachycardia was defined as an HR increase of at least 20 beats per minute (bpm) and HR > 100 bpm after the subject was standing for at least 2 to 4 minutes compared to the HR measured in the supine position. Subjects who experienced orthostatic hypotension and orthostatic tachycardia were identified and summarized by dose group over time and post-baseline overall.

### Physical and neurological examination

The screening physical examination of subjects indicated whether a subject had normal or abnormal findings in each body area (skin/extremities; eye, ears, nose and throat; head/neck, etc.) by dose group. The Visit 9/early termination examination indicated no change, improvement, or worsening compared to baseline in each body system. Summaries by dose group with numbers and percentage of subjects with no change, improvement, or worsening in each body system from the screening visit were provided.

Similar summaries were presented for neurological examinations by visit and overall for each dose group.

### C-SSRS

Results from the C-SSRS assessments were summarized by presenting the number and percentage of subjects with any post-baseline suicidality, any suicidal behavior and type of behavior, and any suicidal ideation and type of ideation for each dose group and overall. Similar summaries were generated by visit. Dose group differences for any post-baseline suicidality, suicidal behavior, and suicidal ideation were evaluated based on Fisher’s Exact tests. Shifts in suicidal ideation from baseline to worst post-baseline result were presented by dose group. Worsening ideation was reflected in shifts from lower to higher values.

## Appendix S2 The Study 046 team

Volodymyr Abramov, Michal Bar, Andrea Bajacekova, Robert Beach, Jay Berke, Perminder Bhatia, Valeny Bitenskyy, Alexis D Boro, Plamen Stoyanov Bozhinow, Oleg Chaban, Andriy Dubenko, Evan Fertig, Edwin A Green Jr., Mercedes P Jacobson, Rosen Stefanov Kalpachki, Sasho Hristov Kastrev, Svitlana Kazakova, David Labiner, Tetyana Litovchenko, Farid Marquez, Patrick E Matoole, Maja Milovanovic, Svitlana Moroz, Evzen Nespor, Joel Oster, Jayoung Pak, Ladislav Pazdera, Tetyana Proskurina, Ralph W Richter, Joanne Rogin, Sofiya Rymsha, Keith Oliver Schluterman, Aashit Shah, Bashir Shihabuddin, Andrii Skrypnikov, Dragoslav Sokic, Mutaz A Tabbaa, Stefan Tsvetanov Tsekov, Viktoryia Verbenko, Monika Zahumenska, Kateryna Zakal.
